# Supplementary material for: Chemical Modification as a Method of Improving Biocompatibility of Carbon Nonwovens
Source: Materials (Basel). 2021 Jun 10;14(12):3198. doi: 10.3390/ma14123198 (PMC8230386; doi:10.3390/ma14123198)
Supplement: Supplementary file 1 [file materials-14-03198-s001.zip › materials-1217083-supplementary.pdf]

materials

Supplementary Materials

Chemical Modification as a Method of Improving Biocompatibility of Carbon Nonwovens

Justyna Frączyk [1,*], Sylwia Magdziarz [1], Ewa Stodolak-Zych [2], Ewa Dzierzkowska [2], Dorota Puchowicz [3], Irena Kamińska [3], Małgorzata Giełdowska [3] and Maciej Boguń [3,*]

[1] Institute of Organic Chemistry, Faculty of Chemistry, Lodz University of Technology, Zeromskiego 116, 90-924 Lodz, Poland; sylwia.magdziarz@dokt.p.lodz.pl
[2] Department of Biomaterials and Composites, Faculty of Materials Science and Ceramics, AGH-UST University of Science and Technology, A. Mickiewicza 30, 30-059 Krakow, Poland; stodolak@agh.edu.pl (E.S.-Z.); dzierzkowska@agh.edu.pl (E.D.)
[3] Łukasiewicz Research Network–Textile Research Institute, Brzezińska 5/15, 92-103 Lodz, Poland; dorota.puchowicz@iw.lukasiewicz.gov.pl (D.P.); irena.kaminska@iw.lukasiewicz.gov.pl (I.K.); malgorzata.gieldowska@iw.lukasiewicz.gov.pl (M.G.)
* Correspondence: justyna.fraczyk@p.lodz.pl (J.F.); maciej.bogun@iw.lukasiewicz.gov.pl (M.B.)

Citation: Frączyk, J.; Magdziarz, S.; Stodolak-Zych, E.; Dzierzkowska, E.; Puchowicz, D.; Kamińska, I.; Giełdowska, M.; Boguń, M. Chemical Modification as a Method of Improving Biocompatibility of Carbon Nonwovens. *Materials* **2021**, *14*, 3198. https://doi.org/10.3390/ma14123198

Academic Editor: Alenka Vesel

Received: 26 April 2021
Accepted: 4 June 2021
Published: 10 June 2021

Publisher's Note: MDPI stays neutral with regard to jurisdictional claims in published maps and institutional affiliations.

Copyright: © 2021 by the authors. Submitted for possible open access publication under the terms and conditions of the Creative Commons Attribution (CC BY) license (http://creativecommons.org/licenses/by/4.0/).

RGD Tripeptide Synthesis

General Procedurę

The peptide was synthesized following a standard solid phase procedure using the Fmoc/tBu protecting group strategy.

Incorporation of the C-Terminal Amino Acid into the 2′-chloro-chlorotrityl Resin

The synthesis was carried out using 1 g of the resin. 2′-Chloro-chlorotrityl resin was swelled in DCM for 1 h. The Fmoc Asp(OtBu)-OH (1.5 fold molar excess with respect to the resin with a 1 mmol/g loading) was dissolved in DCM (10 mL per 1 g of resin) and DIPEA (3 fold molar excess with respect to the amount of resin) was added. The solution was added to the resin and shaken for 1 h. Then, the resin was filtered off and washed with DCM (3 × 3 mL), a mixture of DCM, MeOH and DIPEA 17:2:1 by volume (10 mL), DMF (3 × 3 mL) and DCM (3 × 3 mL).

Determination of the Loading of the 2′-chloro-chlorotrityl Resin

An aliquot (6.11 mg) of the resin was placed in a 10 mL volumetric flask and DCM (0.4 mL) and piperidine (0.4 mL) were added. The suspension was left for 30 min. Then MeOH (1.6 mL) was added and the DCM was made up to the mark. Absorbancję mierzono w zakresie 200–400 nm. As a reference sample, a solution prepared in a manner analogous to the proper sample was used. Based on the absorbance value at 301 nm, the resin density was determined based on the formula:

$$\mathrm{Obs}\left[\frac{\mathrm{mmol}}{\mathrm{g}}\right] = \frac{A_{301}}{0.78 \times m_{zywicy}[\mathrm{mg}]} \tag{S1}$$

The loading of the resin with Fmoc-Asp(OtBu)-OH was 0.348 mmol/g.

Removal of the Fmoc Protecting Group

The Fmoc group was removed using a 25% piperidine solution. The resin was treated with a 25% solution of piperidine in DMF (5 mL per 1 g of resin). The resin was shaken for 15–20 min. The progress of the reaction was monitored by the Kaiser test.

Incorporation of Further Amino Acids Using DMT/NMM/TosO- as a Coupling Reagent

The appropriate blocked amino acid was weighed out with a threefold molar excess to the resin loading. Then DMT/NMM/TosO- was weighed out also using a 3 times molar excess to the resin loading. The amino acid and reagent were dissolved in DMF (approx. 10 mL per 1 g of resin). A measured volume of NMM was added to the solution using a sixfold molar excess to the resin loading. After the reagents had dissolved, the solution was added to the resin (with a free amino group) and shaken depending on the amino acid for 1–4 h. The progress of the reaction was monitored by the Kaiser test (the attachment of the amino acid is evidenced by the lack of navy blue color of the resin grains). When incomplete conversion was found, the coupling reaction was repeated. The solution was then removed and the resin was washed sequentially with DMF (3 × 3 mL) and DCM (3 × 3 mL).

Cleavage of the Peptide from the Resin and Product Isolation

After the peptide was synthesized and the Fmoc group removed from the N-terminal amino acid, the thoroughly dried resin was transferred to a flask and the cleavage mixture (approx. 10 mL per 1 g resin) was added. A cleavage mixture of TFA/H_2O/TIS in a volume ratio of 95/2.5/2.5 was used. The suspension was intensively stirred on a magnetic stirrer for 4 h. The polymer was then filtered off the peptide solution. The solution was evaporated under a stream of nitrogen gas. Diethyl ether (10 mL) was added to the residue. The precipitated crude product was centrifuged and decanted. The product was then dissolved in water and freeze-dried.

Synhesis of Ac-RGD-OH

In the first step of the synthesis, Fmoc-Asp (OtBu)-OH (0.617 g, 1.5 mmol) was attached to 1 g of a 2-chlorotrityl resin according to the general procedure. DIPEA (522 µL, 3 mmol) was used in the reaction. The resin loading was determined in accordance with the general procedure. The calculated resin loading was 0.4 mmol/g. Then, the Fmoc group deprotection was performed on the resin containing the C-terminal amino acid according to the general procedure. For the final peptide synthesis the following amino acids were used: Fmoc-Gly-OH (0.357 g, 1.2 mmol), Fmoc-Arg (Pbf)-OH (0.209 g, 1.2 mmol). DMT/NMM/TosO- (0.496 g, 1.2 mmol) and NMM (660 µL, 3.6 mmol) were used for the each coupling steps. The reactions were carried out according to the general procedure. Fmoc deprotection was performed according to the general procedure using a 25% piperidine solution. In the case of incorporation of the arginine residue, Fmoc deprotection was performed using also a 2% DBU solution. The last step of the reaction was to carry out the acylation reaction of the amino group of the arginine residue. Acetic anhydride (1.02 mL, 10 mmol and DIPEA (3.23 mL, 25 mmol) was used in the reaction. The reaction was carried out for 1 h. After completion of the reaction, the final product was cleaved off according to the general procedure. The final product was obtained with a purity of 90%. Its structure was confirmed by MS, m/z = 389.1921 g/mol, which corresponds to $[M + H]^+$ of the expected product with M = 388.37 g/mol.

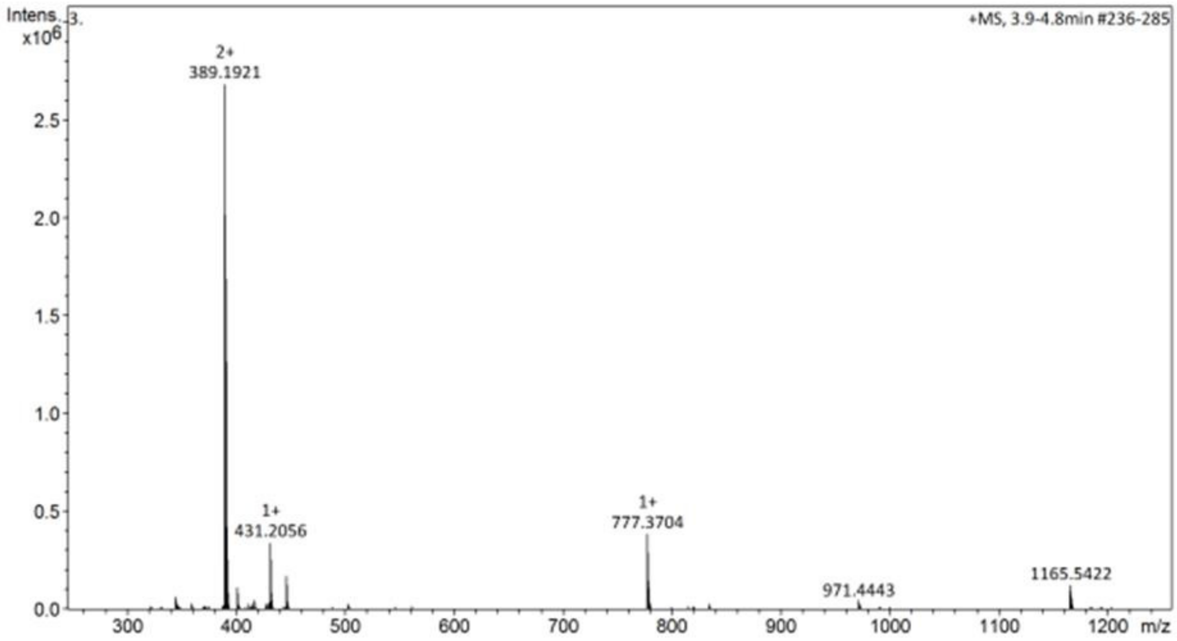

Figure S1. MS spectrum of Ac-RGD-OH.

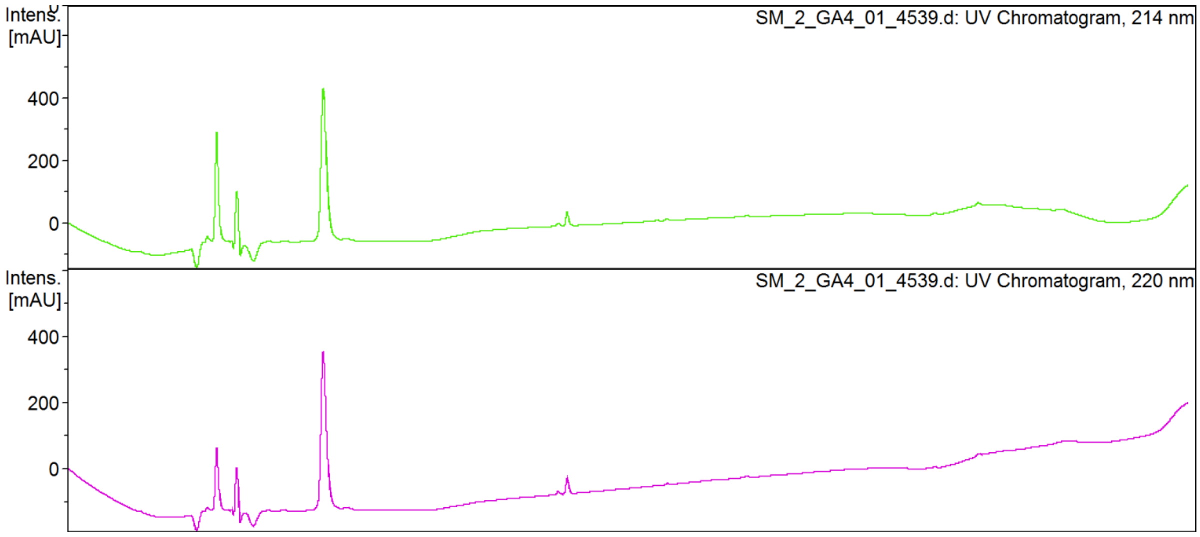

Figure S2. HPLC spectrum of Ac-RGD-OH.

Based on a simple water wettability test of a carbon nonwoven fabric modified with a benzoic acid derivative (**CF-1c**), it was found that the incorporation of a benzoic acid residue on the **CF** nonwoven surface causes a significant increase in hydrophilicity resulting from the presence of the polar COOH group.

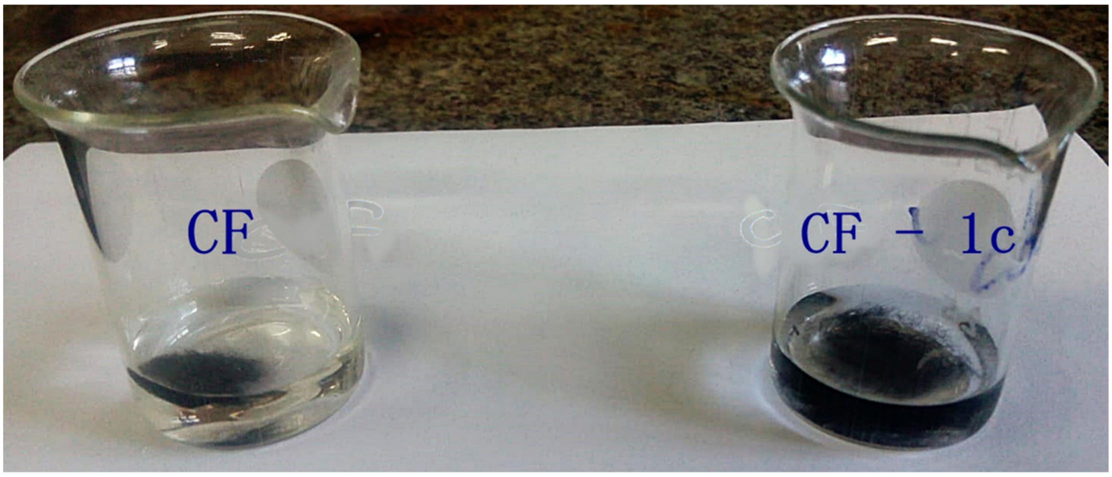

Figure S3. Photos showing the behavior of **CF** nonwoven and **CF-1c** nonwoven after incorporation of the rest of benzoic acid in water. Wettability test.
